# The impact of systemic fungal infection in patients with perforated oesophagus

**DOI:** 10.1308/003588412X13373405388095

**Published:** 2012-05

**Authors:** H Elsayed, H Shaker, I Whittle, S Hussein

**Affiliations:** ^1^Liverpool Heart and Chest Hospital NHS Foundation Trust,UK; ^2^Ain Shams University, Cairo,Egypt

**Keywords:** Perforated oesophagus, Fungal infection, Antifungal

## Abstract

**INTRODUCTION:**

Perforated oesophagus is a surgical emergency with significant morbidity and mortality. Systemic fungal infection represents a poor response to the magnitude of the insult, which adds significantly to the risk of morbidity and mortality in these patients. We reviewed our experience with this group of patients over a six-year period in a tertiary referral centre.

**METHODS:**

A retrospective clinical review was conducted of patients who were admitted following a ruptured oesophagus over a period of six years (January 2002 – January 2008).

**RESULTS:**

We had 27 admissions (18 men and 9 women) following an isolated perforated oesophagus to our unit. The median patient age was 65 years (range: 22–87 years). The majority (*n*=24,89%) presented with spontaneous perforations (Boerhaave’s syndrome) and three (11%) were iatrogenic. Fungal organisms, predominantly *Candida albicans*, were positively cultured in pleural or blood samples in 16 (59%) of the 27 patients. Fourteen patients grew yeasts within the first seven days while two showed a delayed growth after ten days. Overall mortality was 5 out of 27 patients (19%). There was no mortality among the group that did not grow yeasts in their blood/pleural fluid while mortality was 31% (5/16) in the group with systemic fungal infection (*p*<0.001). A positive fungal culture was also associated with increase ventilation time, intensive care unit stay and inpatient hospital stay but not an increased rate of complications.

**CONCLUSIONS:**

Systemic fungal infection in patients with a ruptured oesophagus affects a significant proportion of these patients and carries a poor prognosis despite advanced critical care interventions. It may represent a general marker of poor host response to a major insult but can add to mortality and morbidity. It is worth considering adding antifungal therapy empirically at an early stage to antimicrobials in patients with an established diagnosis of a perforated oesophagus.

Perforation of the oesophagus is a serious condition that is associated with significantly high morbidity and mortality despite advances in diagnostics, surgery and critical care.^1^ The aetiology of oesophageal perforations managed in most centres in Europe and the US is commonly of two types: iatrogenic and spontaneous perforation (Boerhaave’s syndrome).Less common causes include perforations secondary to malignancy or trauma.^1^

Despite a large number of studies, controversy still exists about many aspects of the management of oesophageal perforations including the role of conservative management, primary repair, defunctioning of the oesophagus, managing patients with delayed presentation and the best combination of antisepsis medication as these patients commonly present with profound sepsis.

*Candida* represents a commensal fungus of different mucous membranes in healthy individuals. The oesophagus is colonised in as much as 25% of normal individuals.^2^ However, invasive infections causing extensive tissue necrosis and ulceration occur predominantly in immuno-compromised patients after major surgical procedures or prolonged intensive care unit stays and also in patients with organ transplantations.^3^ It has only been partially elucidated which factors may contribute to promote progression from simple colonisation to invasive disease.^4^

Patients with a perforated oesophagus have several factors that will predispose them to systemic fungal infection, which may have an impact on their outcome. In this report, we observe the incidence of systemic fungal infection in patients with a perforated oesophagus in a tertiary referral centre and its impact on patient prognosis.

## Methods

A retrospective review was conducted for all patients admitted to our institution with oesophageal perforation over a six-year period between January 2002 and January 2008.Patients with oesophageal leaks secondary to oesophageal or thoracic surgery were excluded as they represent a different cohort of patients.

The hospital and theatre databases as well as clinical notes were reviewed for information concerning clinical presentation, initial management, clinical course and mortality. Data gathered included aetiology, method of and time to diagnosis, co-morbidities, patients’ clinical condition on admission and type of initial management (operative or non-operative).

Early diagnosis was defined as confirmation of oesophageal perforation less than 24 hours since the presumed onset of symptoms. Late diagnosis indicated this was any time after 24 hours. Major co-morbidities were defined as ischaemic heart disease, valvular heart disease, atrial fibrillation, diabetes, chronic obstructive pulmonary disease, renal failure or underlying malignancy. Gastro-oesophageal co-morbidity included oesophagitis, gastro-oesophageal reflux disease, peptic ulcer disease or benign strictures. Surgical management consisted of primary repair or oesophagectomy and diversion.

On admission, all patients were started on ceftriaxone and metronidazole from their referring centre. Patients who were overtly septic and required organ support were put on broad-spectrum antibiotics after discussion with a microbiology consultant (usually meropenem 1g three times daily). Post-operatively, cultures were sent for microbiological analysis from all lines (cannulas, central lines, arterial lines, urinary catheters, Swan-Ganz catheters [if applicable] and pleural fluid). Blood cultures were sent for patients showing persistent sepsis (high white cell count, high C-reactive protein levels or requiring organ support). Antibiotics or antifungals were started/modified accordingly. Antifungal medication was only commenced on isolating fungal species (through cultures) in patients with a clinical picture of sepsis. Fluconazole 400mg/day was the treatment of choice for fungal infection.

Data were also collected on critical care and management, post-operative progress and resumption of oral intake, post-operative intensive care unit (ICU) stay, length of hospital stay, inpatient major complications (ventilatory/circulatory support), other complications (chest infection, wound infection, renal failure, gastrointestinal [GI] bleed and pericardial effusion)and inpatient mortality. Chest infection was defined as the presence of parenchymal consolidation, a productive cough and a raised white cell count on a full blood count.

Statistical tests used for univariate analyses included Fisher’s exact test, the Mann–Whitney U test and Student’s t-test.In all cases, a *p*-value of <0.05 was considered significant. All statistical analyses were performed using SAS®version 8.2 (SAS, Cary, NC, US).

## Results

Twenty-eight patients with oesophageal perforation were identified from our database. One patient was excluded as the initial management of the perforation took place at a hospital in Italy before being transferred to our care after stabilisation, leaving 27 patients for review. There were 18 men and 9 women with a median age of 65 years (range: 22–87 years).Seven patients had major co-morbidities (mainly cardiovascular) and seven had gastro-oesophageal co-morbidities while two had both (Table 1). Three patients had a history of heavy alcohol use during their perforation (all spontaneous).

**Table 1:** Characteristics of patients managed for oesophageal perforation

	Number of patients
*Sex*	
Male	18
Female	9
Median age (range)	65 years (22–87 years)
*Gastro-oesophageal co-morbidities*	
Hiatus hernia	1
Gastro-oesophageal reflux disease	4
with Barrett’s oesophagus	1
Peptic ulcer disease	2
Benign oesophageal stricture	1
*Major co-morbidities*	
Ischaemic heart disease	6
Chronic airways disease	1
Stroke	1
*Smoking and alcohol use*	
Smoker	11
Non-smoker	16
Heavy alcohol use	3
Moderate alcohol use	11
No alcohol	13
*Aetiology*	
Spontaneous	24
Iatrogenic (gastroscopy)	2
Iatrogenic (nasogastric tube insertion)	1
*Side of perforation*	
Right	12
Left	15

### Critical care support

Of the 27 patients referred, 23 were admitted electively to our critical care unit on admission. Six patients required both haemodynamic support and mechanical ventilation on admission to our unit. Eight patients required mechanical ventilation or haemodynamic support alone while the remaining nine only required fluid resuscitation or were stable. Of the 14 patients who required inotropic or ventilatory support on admission, 11 (78%) survived to discharge. Of the remaining 13 patients who were stable on admission, just over 84% survived. This difference was not statistically significant (*p*>0.5).

The four patients who were not admitted to the critical care unit were completely stable from diagnosis to arrival at our hospital. Three of these patients were managed surgically and so were only admitted to the critical care unit post-operatively.

### Type of repair

Twenty-three patients were taken to theatre and assessed for suitability for primary repair of their perforation. Thirteen were approached via a right thoracotomy while ten were approached from the left side. Primary repair of the perforation was attempted in 21 patients (77%).Repair of the perforation consisted of two-layer interrupted suturing of the mucosal and muscularis layers with absorbable sutures after debridement and excision of necrotic and inflamed tissue. Where possible, this was reinforced by a pedicled intercostal muscle flap sutured over the repaired defect. Primary repair was not attempted in 2 (7%) of the surgical cases owing to the state of intrathoracic sepsis where an oesophagectomy and diversion was performed. Reanastomosis was performed at a later stage.

**Figure 1:**
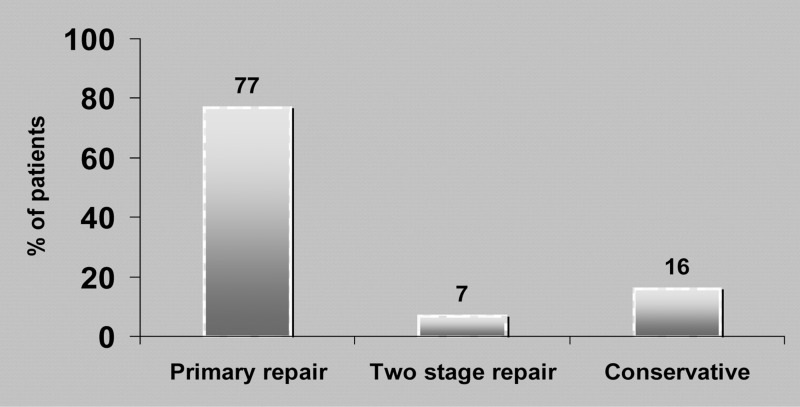
Different treatment modalities used for patients with oesophageal perforation

Non-operative management was used in four patients (16%).Two were patients with small, contained leaks who showed no signs of a systemic inflammatory response or sepsis and two were deemed a high anaesthetic risk (Fig 1).

### Microbiological results

All patients were treated with broad spectrum antibiotics on admission. Positive bacteriology was obtained in over 75% of patients from pleural, mediastinal or blood samples, which guided further treatment. The three most common organisms cultured were enterococci, coagulase negative *Staphylococcus aureus*, and lactose fermenting and non-lactose fermenting coliforms.

Fungal organisms, namely *Candida albicans*, were positively cultured in pleural or blood samples in 14 of the 27 patients within the first 7 days. Twelve of these patients received antifungal treatment. Two patients grew fungal organisms more than ten days following perforation, one of which was from a central venous line.

Two patients who did not have yeasts cultured from any of their microbiological samples were given antifungal therapy owing to persistent signs of sepsis despite broad spectrum antibiotics. There was no difference between the fungal/no fungal infection group regarding age, time to diagnosis, type of repair or co-morbidities (Table 2).Among these factors, a delay in diagnosis (>24 hours) was the factor associated with a significantly higher mortality (40% vs 6.2%, *p*=0.047).

**Table 2:** Comparison between the fungal/no fungal infection groups regarding pre-operative and operative intervention factors

	Fungal organisms cultured	No fungal organisms cultured	P-value
Number of patients	16	11	
Mean age (years)	65.5	64	0.8
Late diagnosis (>24 hrs)	5 (31%)	6 (54%)	0.08
Primary surgical repair	12 (75%)	9 (80%)	0.6
Co-morbidities	8 (57%)	7 (63%)	0.12

### Impact of fungal infection

Overall mortality was 19% (5/27). All five non-survivors had yeasts cultured from one or more specimens during their hospital stay. They all had antifungal therapy commenced after diagnosis of infection until time of death. There was no mortality among the group who did not grow yeasts in their blood/pleural fluid while mortality was 31% (5/16) in the group with cultured fungal infection (*p*<0.001). In all cases, the cultures were grown in the first seven days from pleural or mediastinal samples either at operation or from a chest drain.

**Figure 2:**
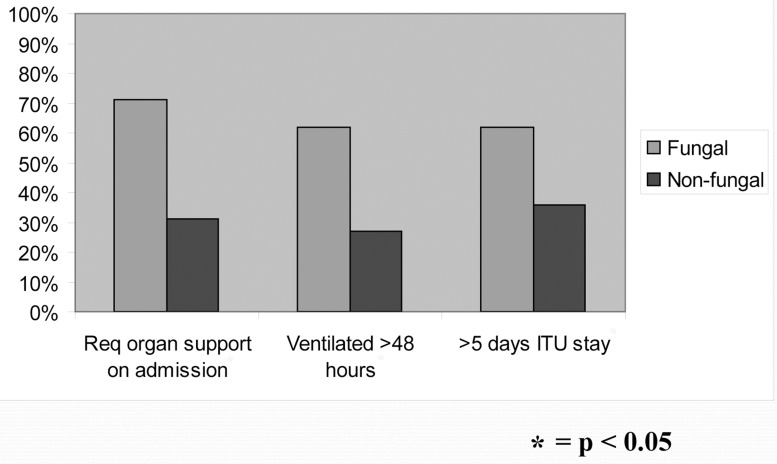
Comparison between the fungal/no fungal infection groups regarding pre-operative requirement of organ support on admission, duration of ventilation and length of stay on intensive care unit (ICU)

The mean hospital stay was 31 days (range: 13–80 days). A positive fungal culture was also associated with increased pre-operative requirement of organ support (*p*=0.001), ICU stay (*p*=0.03) and inpatient hospital stay (*p*=0.02) (Fig 2) but not an increased rate of other complications (wound infection, chest infection, renal failure, GI bleed or pericardial effusion) (*p*>0.05).

## Discussion

To our knowledge, this is the largest study in the English literature that examines the effect of fungal infection on patients with a ruptured oesophagus. Candidaemia has been recognised increasingly as a cause of septicaemia, occasionally outnumbering all Gram negative bacilli sepsis.^5^ A number of factors have been reported to predispose patients to fungal infection. Impaired T cell function owing to factors such as high-dose glucocorticoid therapy, chemotherapy or acquired immunodeficiency syndrome as well as depressed neutrophil number or function from causes such as haematological malignancies and chemotherapy may increase the risk of fungal infection.^6^ The use of multiple broad-spectrum antimicrobial agents in critically ill patients may alter the normal flora in the GI tract and precipitate fungal infection, and is an important factor in the increasing occurrence of fungal infection in patients with a ruptured oesophagus, who are usually on long-term antimicrobial therapy.^6^

Disruption of the normal GI flora may allow overgrowth of *Candida* spp, which colonise the oesophagus and can then translocate to the bloodstream. In addition, because *Candida* spp commonly colonise the skin, any break in the epithelial barrier could act as a portal for invasion. The increasing use of hyperalimentation and invasive devices including central vascular catheters, urinary catheters and chest tubes therefore also contributes to the development of fungal infections in patients with a ruptured oesophagus.^7^ Because of drainage tube insertion and damage to the normal mucosal barrier, chest and abdominal surgery can also put patients at risk of fungal infection.^8^

As with any alimentary tract perforation, antimicrobial therapy plays a key role in the management of the ensuing bacterial contamination of adjacent cavities (eg mediastinum) by commensal organisms that takes place following oesophageal perforations. The initial use of broad-spectrum antibiotics should be followed by targeted therapy guided by culture and sensitivities from pleural, mediastinal and blood samples where available.

Despite *Candida* spp being an oesophageal commensal^2,9^ and a common source of non-perforation related oesophageal infections,^10^ little has been reported on the role of species of yeasts in sepsis linked to oesophageal perforations. Sepsis is a major cause of death after oesophageal perforations. Three out of the five patients who died in our series died from multiorgan failure secondary to sepsis. A few case reports have reported candidal sepsis^11^ and candidal mediastinitis^12,13^ as serious complications of oesophageal perforation, the latter being more fatal.

The morbidity and mortality impact of fungal infection in patients with a ruptured oesophagus was evident in our study. Among our patients, a positive fungal culture was associated with increased ventilation time, ICU stay and inpatient hospital stay. In addition, all patients who died had positive fungal cultures and the mortality rate among those with positive fungal cultures was 35% compared with 19% among the whole group.

Delay in diagnosis has been shown to be a major contributor in several series.^14,15^ This may represent the fact that a longer period without treatment following a perforation means further mediastinal contamination, inflammation and subsequent development of systemic sepsis. There was no statistically significant difference in our series in time to diagnosis between the group with a fungal infection and the group with no signs of fungal sepsis.

At our centre, primary repair of oesophageal perforations is always favoured as it allows drainage of infection as well as debridement of necrotic and inflamed tissue. Furthermore, it permits full exposure of the mucosal defect, and approximation of the mucosal and submucosal edges, resulting in optimal conditions for adequate healing. Findings from several series focusing mainly on spontaneous perforations have shown that primary repair offers the best chance of survival, regardless of presentation time.^16,17^ It is considered to be the best treatment option for uncontained thoracic perforations of an otherwise healthy oesophagus, with other treatment modalities being the exception and reserved for select cases. There was no difference in our series regarding incidence of primary repair between the fungal infection/no fungal infection groups. Additionally, there was no difference between the groups regarding patient co-morbidities.

The choice of a suitable antifungal therapy would be guided mainly by a balance between its efficacy, potential hazards and cost. Fluconazole is considered the first line prophylaxis and therapy among patients who have not been treated previously with azoles (as prophylaxis), who have a mild to moderate illness and who are not at high risk of *Candida krusei* or *Candida glabrata* (elderly patients, patients with diabetes and malignancy). Fluconazole was administered in most trials at an adult dosage of 400mg/day or less.^18^ In a meta-analysis, Gafter-Gvili *et al* found no significant difference in mortality between fluconazole and amphotericin B but noted a significantly higher risk of microbiological failure in the fluconazole arm and of nephrotoxicity in the amphotericin B arm.^19^

Candidiasis that fails to respond to treatment has been reported increasingly, particularly among patients who have not benefited from fluconazole and other azole drugs. This is due partly to the widespread, long-term use of azoles for treating and preventing candidiasis. Other factors include treatment with antituberculosis drugs, treatment with ciprofloxacin and CD4+ cell counts below 50/µl. Resistance to azole drugs has often required using amphotericin B. While potent and effective, amphotericin B is toxic, especially to the kidney.^18,19^

Echinocandins are a new class of antifungal agents for the treatment of *Candida* spp. They are non-competitive inhibitors of the synthesis of β-1,3-glucan and a constituent of the *Candida* cell wall. Their administration schedule is convenient (once daily) and their activity is fungicidal against all *Candida* spp.^20^ The comparison of echinocandins with other antifungal agents demonstrated no inferiority and a better safety profile for echinocandins. Lower risks of treatment and microbiological failure were observed with anidulafungin when compared with fluconazole. No significant differences in mortality were found and no significant differences in efficacy or adverse events were observed among the different echinocandins.^19^

The Infectious Diseases Society of America treatment guidelines allow the practitioner to choose between fluconazole, an amphotericin B preparation, combination therapy with fluconazole plus amphotericin B or caspofungin.^21^ Echinocandins have a major role in the treatment of invasive candidiasis. The meta-analysis by Gafter-Gvili *et al* also demonstrated superior efficacy for anidulafungin versus fluconazole and comparable efficacy for caspofungin and micafungin versus amphotericin B formulations.^19^ Furthermore, echinocandins have a better safety profile than both azoles and polyenes. For empirical treatment of candidaemia, therefore, echinocandins may be considered as the first line treatment. Liposomal amphotericin B is an equally good alternative. However, one cannot ignore the high cost of both these options. In locations where cost and availability present barriers to their use, amphotericin B and fluconazole, which has a comparable mortality to amphotericin B, are also effective alternatives.

In the literature, there is only one German series that had a big number of patients studying the effect of fungal sepsis following a ruptured oesophagus. In their series of 58 patients,Jungbluth *et al* found that the mortality rate was 50% among patients with positive fungal cultures compared with 28% overall.^22^ The mean time spent in the ICU was greater in the group of patients with positive fungal cultures and this group was also more likely to develop septic complications such as mediastinitis, pneumonia and empyema. Similar to our findings, they noted that there was no difference between the fungal and non-fungal groups in terms of time to diagnosis, aetiology or type of management (conservative vs surgical).

Controversy exists regarding the use antimycotic therapy in oesophageal perforations. Jungbluth *et al* recommend only targeted therapy,^22^ despite the high risk of complications, while Bauer *et al* advocate empirical therapy for all cases although their recommendation is only based on a report of two cases.^11^

With our large series of patients, we feel a case may exist for putting all patients with confirmed oesophageal perforation on empirical antimycotic therapy for two main reasons. First, almost all cases of candidal cultures were grown from samples taken early in the patients’ stay and not cultured after a period of treatment, suggesting the development of local candidal sepsis soon after oesophageal perforation. *Candida* colonisation is known to be significantly higher in patients with benign oesophageal disease, itself a risk factor for perforation according to some, so the proportion of colonised patients presenting with perforations may be higher than in the average population.^23^ Second, the incidence of systemic candidaemia in critically ill patients (of any cause) has increased, and is a significant cause of mortality and prolonged stay on the ICU.

We admit that fungal infection cannot be considered a risk factor on its own as it usually reflects a consequence of poor response to treatment and is frequently a part of multisystem failure in a sick patient after a perforated oesophagus. Although the five patients in our series who died did have antifungal therapy this was at a later stage, after diagnosis of an established fungal infection. We believe that systemic fungal infection in patients with a perforated oesophagus can be an extra risk in those already very unwell patients. Adding an antifungal at an early stage at the time of diagnosis may help reduce aggravation of the risk by reducing the chances of a superimposed systemic fungal infection.

Some authors have concluded that antifungal prophylaxis could reduce mortality by 25% in non-neutropaenic critically ill patients and should be given prophylactically to patients at increased risk of invasive fungal infections.^24^ Patients with oesophageal perforation, the majority of whom are managed initially on critical care units, have several factors that increase their risk of secondary candidal infection including prolonged antibiotic use, surgery and being on total parental nutrition as well as a possible higher rate of candidal colonisation. As a result, this makes them ideal candidates for empirical antifungal therapy from diagnosis. This is the routine practice in our hospital now.

Until a randomised study comparing administration of antifungal versus no antifungal therapy proves empirically that there is no benefit of adding this medication, antifungal prophylaxis should be standard in patients with a ruptured oesophagus once diagnosed. We appreciate the limitation of this study in terms of the number of patients but as a ruptured oesophagus is a rare presentation, it would be difficult to have a randomised study with a large number of patients.

## Conclusions

Systemic fungal infection affects a significant proportion of patients with a ruptured oesophagus and carries a poor prognosis despite advanced critical care interventions. It may represent a general marker of poor host response to a major insult but can add to mortality and morbidity. It is worth considering adding antifungal therapy empirically at an early stage to antimicrobials in patients with an established diagnosis of a perforated oesophagus.
